# Interfacial tension and wettability alteration during hydrogen and carbon dioxide storage in depleted gas reservoirs

**DOI:** 10.1038/s41598-024-62458-5

**Published:** 2024-05-21

**Authors:** Mohammad Rasool Dehghani, Seyede Fatemeh Ghazi, Yousef Kazemzadeh

**Affiliations:** Department of Petroleum Engineering, Faculty of Petroleum, Gas, and Petrochemical Engineering, Persian Gulf University, Bushehr, Iran

**Keywords:** Interfacial tension, Wettability alteration, Carbon dioxide storage, Depleted gas reservoirs, Engineering, Chemical engineering

## Abstract

The storage of CO_2_ and hydrogen within depleted gas and oil reservoirs holds immense potential for mitigating greenhouse gas emissions and advancing renewable energy initiatives. However, achieving effective storage necessitates a thorough comprehension of the dynamic interplay between interfacial tension and wettability alteration under varying conditions. This comprehensive review investigates the multifaceted influence of several critical parameters on the alterations of IFT and wettability during the injection and storage of CO_2_ and hydrogen. Through a meticulous analysis of pressure, temperature, treatment duration, pH levels, the presence of nanoparticles, organic acids, anionic surfactants, and rock characteristics, this review elucidates the intricate mechanisms governing the changes in IFT and wettability within reservoir environments. By synthesizing recent experimental and theoretical advancements, this review aims to provide a holistic understanding of the processes underlying IFT and wettability alteration, thereby facilitating the optimization of storage efficiency and the long-term viability of depleted reservoirs as carbon capture and storage or hydrogen storage solutions. The insights gleaned from this analysis offer invaluable guidance for researchers, engineers, and policymakers engaged in harnessing the potential of depleted reservoirs for sustainable energy solutions and environmental conservation. This synthesis of knowledge serves as a foundational resource for future research endeavors aimed at enhancing the efficacy and reliability of CO_2_ and hydrogen storage in depleted reservoirs.

## Introduction

While the emission of greenhouse gases (GHG) caused by human activities remains the primary driver of global warming and climate change, it is rapidly increasing^1,2^, The implementation of appropriate measures to reduce global temperature and its associated human impacts has become increasingly vital. Carbon dioxide resulting from the burning of fossil fuels in power plants and energy industries constitutes approximately three-fourths of greenhouse gases^3,4^. However, alongside the pivotal role of hydrocarbons in energy production, efforts to curb the resulting greenhouse gas emissions and the promotion of clean energy have gained significant traction^5^. Carbon Capture and Storage (CCS) is an essential technique, with contemporary initiatives forecasted to diminish CO_2_ discharges by a remarkable 30 million tonnes annually. In addition, CCS alone could contribute almost 20% to reducing greenhouse gas emissions by 2050 and the removal of CCS would increase global costs for achieving emission reduction targets by as much as 70%^6^. Net CO_2_ emission reductions, significant storage capacity, longterm operational separation of CO_2_ from the atmosphere, cost-effectiveness, and low environmental impact mitigation are crucial features of carbon dioxide storage options^7^. Moreover, the employment of hydrogen (H_2_) as an eco-friendly energy origin has also drawn extensive international focus from the global energy community^8^. The energy density of hydrogen is 3.2 times less than that of natural gas and 2700 times less than that of gasoline. Therefore, it can be inferred that hydrogen can serve as an energy carrier instead of a source of energy. This implies that hydrogen can store and deliver energy in a usable form. The higher hydrogen efficiency (60% compared to 22% for gasoline or 45% for diesel) contributes to the improvement of energy efficiency for the use of clean energy in the future. A number of methodologies have been proposed for the creation and storage of hydrogen in underground caverns. As a result, underground hydrogen storage (UHS) presents a hopeful technique for enclosing H_2_ in geological formations beneath the earth's surface, allowing for its retrieval when necessary^9–11^.

Given the importance of interfacial tension (IFT) and wettability in determining the storage capacity, trapping, and recovery of carbon dioxide and hydrogen, this study for the first time will comprehensively investigate the changes in these two parameters during gas storage, considering various factors such as temperature, pressure, salinity, and different nanoparticles based on previous studies. The results of this research can help to understand the interactions during gas storage and lead to improving the efficiency of these operations.

## Gas storage in depleted reservoirs

Investigating the broad features, structure and efficiency of hydrocarbon reservoirs as an environment for storing hydrocarbons becomes important after the end of the extraction period and converting them into a gas storage device.

To expand, oil or gas reserves, also referred to as hydrocarbon reservoirs, are geological structures that have gone through different diagenetic processes including formation from a source rock, subsequent movement, and maturation to eventually function as a storage medium for hydrocarbons. Predominantly, these formations are sealed by a non-permeable cap rock, which frequently benefits from the support of the underlying water boundaries. When a gas field approaches the end of its effective extraction period, it often transitions into a storage site for gas. A gas reservoir that has been depleted or is nearing depletion typically exhibits reduced pressure and higher water saturation in the space formerly occupied by the gas, brought about by variations in aquifer levels. Consequently, the level of gas saturation behind the advancing water front varies from a minimum—associated with the residual gas saturation close to the original gas and water contact point—to a maximum near the area where gas and water are actively contacting each other^12^. In simpler terms, one could conceptualize a depleted gas reservoir as being a component of an aquifer system—or more specifically, geological traps—where water remains only in minimal quantities within the pores, with gas constituting the predominant substance.When a gas field approaches the tail end of its production phase, it’s often repurposed into a storage area for gas. As it gets depleted, the reservoir’s characteristics change; it’s marked by diminished pressure and an increase in water presence in the areas that gas once filled. The water movement alters the levels of gas saturation, creating a spectrum from the lowest at the initial gas–water interface to the highest around the proximity of this contact zone. Thus, a gas field at the brink of exhaustion can be seen as a segment within a water-bearing geological formation, one where gas predominantly fills the pore spaces, despite the presence of limited water volumes. Depleted gas fields can be utilized for gas storage as their impermeability (resistance) has been proven over geological time^13^. Until now, these types of reservoirs have been well identified due to their geological characteristics, trap integrity, and prior exploration, development, and production activities that have been extensively researched. They are commonly used for natural gas storage^14^. The inventory of underground gas storage is composed of two types of gas: working gas and cushion gas, as we have seen^15,16^. In this scenario, the operational gas H_2_ is conventionally introduced and removed from the storage as needed. Meanwhile, the cushion or base gases, such as CO_2_, CH_4_, N_2_, and even H_2_, are maintained in the facility to ensure pressure levels are upheld through the compression and expansion processes during injection and extraction cycles^17^. Furthermore, it prevents water ingress for optimal storage space. The inventory of subterranean gas storage is typically made up of working gas and what’s known as cushion or base gas, as studies^15,16^ have shown. In instances where H_2_ serves as the working gas, it is regularly cycled in and out of the storage system in response to demand fluctuations. Meanwhile, the cushion gas, which might be CO_2_, CH_4_, N_2_, or even H_2_, stays consistently within the storage system. Its main function is to preserve the necessary pressure by means of compression and decompression during the cycles of injection and extraction^17^. This enduring gas presence also plays a key role in safeguarding against the infiltration of water, thereby optimizing the available storage capacity^18^ and reducing the impact of injected gas impurities. Throughout the operational phase, hydrogen that is pumped into the reservoir interacts with and gradually moves the pre-existing fluids, which include brine and any leftover gas, found inside the pore spaces. This hydrogen then extends and permeates across a low-permeability barrier which is designed to contain fluids^8^. Due to density variations between the injected H_2_ and the native fluid, which occur with pressure increase, the fluid is pushed downwards or laterally to create storage space. As pressure increases steadily during the injection, an interface between the fluid and gas develops. This newly-created boundary, separating H_2_ from the brine and gas within the pores, can impede the extraction process, posing particular challenges for short-duration storage scenarios^19,20^. Since this phase is miscible with the injected hydrogen (H_2_), it is logical to anticipate that in the initial cycles of hydrogen injection and withdrawal, the recovered gas will contain a certain proportion of this mixture, which is expected to decrease as the number of storage cycles increases. But it remains unclear how much native gas mixing is taking place, for example, CH_4_, CO_2_, N_2,_ and the injection gas interaction with gaseous phases. While previous experience in the storage of natural gas has demonstrated that pistonlike behavior and limited mixing between injected gases and natural gas occurs, it is not known whether this applies to H_2_ during injection into a tank. As H_2_ is introduced under increasing pressure, its density differs from that of the indigenous fluid, causing the resident fluid to be displaced downward or sideways, thus carving out space for storage. As the pressure continues to escalate, an interface of fluid and gas takes shape in the course of the injection phase. This emerging boundary within the pore spaces, where H_2_ meets brine or gas, can impede the retrieval of gas, an issue that’s particularly pronounced in short-duration storage scenarios^19,20^. Since the phase encountered will blend with the H_2_, one could foresee that the initial cycles of injecting and drawing H_2_ would result in the extraction of a diminishing portion of this mixed gas as the storage cycles progress. The extent to which the originally present gases (e.g., CH_4_, CO_2_, N_2_) will mix with the H_2_, and how the gas and liquid phases will interact, remains a subject shrouded in uncertainty. Prior knowledge gained from storing natural gas suggests a piston-like movement with minimal intermingling of the injected and native gases, but whether this pattern will be replicated with H_2_ injections into the reservoir is yet to be determined^21^.

## Hydrogen and carbon dioxide storage

The study of various characteristics and chemical and physical properties of hydrogen and carbon dioxide on storage and its type in geological structures is investigated due to its wide effects.

### Carbon dioxide storage

In geological settings, carbon dioxide can be confined through a range of physical and chemical capture methods, undergoing transformations in its physical state due to the unique pressures and temperatures found beneath the Earth's surface. Under conditions prevalent at the surface, CO_2_ is in gaseous form and possesses a slightly higher density than air, at 1.872 kg/m^3^.

#### Physical trapping

The physical capture of CO_2_ happens when CO_2_ is confined as a supercritical fluid or a stabilized gas, and it is a process that depends on volume. There are two forms of physical trapping:

- Static trapping means that CO_2_ is blocked by a low-permeability layer or a man-made barrier, and can only flow if there is an opening.
- Residual trapping means that CO_2_ is stuck in the pores of the rock, and cannot flow even if there is an opening, because of the surface tension between CO_2_ and water.

#### Chemical trapping

Chemical trapping occurs when CO_2_ is absorbed onto organic matter present in coal and shale (adsorption trapping), or dissolved in subsurface fluids (solubility and ion trapping), and may react with the rock matrix (mineral trapping)^22,23^.

#### CCS

CCS involves a wide range of processes for capturing, separating, transporting, storing, and monitoring CO_2_ emissions. However, due to the large amount of carbon that needs to be stored and the fact that the pure CO_2_ is buoyant, whether gas or supercritical and has a tendency to migrate, the storage of captured carbon during CCS efforts presents challenges. It can reoccur if it's not sufficiently preserved. There are currently two deep subsurface storage options being examined. The first approach is to inject captured carbon into sediment basins where CO_2_ can be physically trapped under impermeable rock, preventing it from being transferred onto the surface. Ensuring that the system's caprock is sufficiently impermeable to ensure longterm storage without leakage is a key requirement for such storage. On the other hand, the captured carbon can be stored by injection into reactive rocks such as basalts or ultramafic rocks, which lead to the trapping of carbon in stable carbonate minerals. The injected carbon is permanently sequestered and poses minimal risk of returning to the atmosphere by stimulating the mineralization of the injected CO_2_ in carbonate minerals such as calcite, dolomite, and magnesite by injecting it into reactive host rocks^24^.

Recently, ionic liquids (ILs) have been proposed as a potential replacement solvent for amines in the carbon capture processes. Strong ion-ion interactions for ILs lead to negligible evaporation at the ambient conditions. Other appealing properties of ILs are their high thermal stability, large electrochemical window, and ability to dissolve compounds with various polarities^25^.

One other materials that can be used for carbon capture operations are liquid polymers. Using these materials for CCS comes with advantages such as:

- Can be obtained from natural products and by-products from industries
- Biodegradable
- Reasonable permittivity and selectivity
- Easy to utilize in industries
- Can be used by itself or as an auxiliary component alongside ionic liquids

And some disadvantages like:

- Cannot be used for intensive carbon capture
- Requires higher pressures for optimal performance^26^.

### CO2 separation options

Potential CO_2_ separation options include groundwater storage, ocean depths, and mineral carbonation as part of CCS^23^. In the realm of Carbon Capture and Storage (CCS), plausible techniques for the sequestration of CO_2_ encompass a variety of strategies such as subterranean geological storages, deep oceanic disposals, and mineral carbonation processes^23^. The geological storage category encompasses a range of alternatives including saline formations, depleted oil and gas fields, coal deposits that are not viable for mining, hydrate formations for CO_2_ capture, and pioneering geothermal energy systems powered by CO_2_^27–29^.

#### Underground geological CO_2_ storage

Geological storage is the best way to deposit CO_2_ underground for CCS. It is also better than mineral carbonation or ocean acidification because of cost, location, safety, and environmental impact.

##### Saline aquifers

Storing CO_2_ in saline aquifers is effective due to its high storage potential and limited conflicting uses. However, the lack of infrastructure makes many such aquifers economically unattractive for water storage^29–37^.

##### Depleted oil and gas reservoirs

Storing CO_2_ in depleted oil and gas reservoirs is highly effective due to existing infrastructure and prior assessment for storage capacity. Additionally, gas injection techniques used in the oil and gas industry can be applied for CO_2_ storage^38–43^.

##### Unmineable coal seams

Storing CO_2_ in unmineable coal seams is a viable option due to the presence of fractures and pores in the coal matrix, allowing for gas absorption. The injected CO_2_ replaces methane, leading to increased production and storage of significant CO_2_ amounts, while enhancing profitability in coal bed methane operations. Successful implementation of Enhanced Coal Bed Methane (ECBM) requires specific technical criteria outlined by IEAGHG, including reservoir homogeneity, minimal faulting/fracturing, optimal depth range, concentrated coal seam geometry, and sufficient permeability^44–47^.

##### Basalt formations

Basalt rocks constitute approximately 8% of the Earth's continents and comprise a significant portion of the ocean floor, indicating their enormous potential for CO_2_ storage^48^. Primary favorable characteristics of these substances regarding their carbon-capture capabilities encompass their significant reactive nature along with the plentiful presence of bivalent metal ions within these minerals, which may have the capacity to trap CO_2_ over extended geological periods^49^.

##### Hydrate storage of CO_2_ within the subsurface environment

Subsurface storage of CO_2_ as hydrates is a promising option, utilizing CO_2_ hydrates to trap CO_2_ molecules within a network of water molecules. It can be formed quickly in the presence of water and good pressure/temperature conditions and may offer self-sealing properties in some cases. However, its use in certain environments is restricted by stability limitations at moderate pressures and temperatures below 10 °C, such as in shallow deposits under freezing waters and dense permafrost without nearby CO_2_ sources^28,50,51^.

##### CO_2_-based enhanced geothermal systems

CO_2_, with its superior physical characteristics including lower viscosity and greater clarity, can efficiently transfer heat and be used for geothermal energy generation. It can penetrate rock masses and serve as a working fluid in enhanced geothermal systems due to its low viscosity. Unlike water, the use of CO_2_ in enhanced geothermal systems doesn't result in fluid loss, which has economic consequences. Additionally, CO_2_ -based EGS offers the potential for geological storage of CO_2_ underground, providing an additional benefit^27,52–55^.

#### Deep ocean storage

An intentional injection of CO_2_ into deep ocean waters is a potential alternative strategy for human CO_2_ separation. The average depth of the oceans is 3.8 km, covering 70% of Earth's surface^56^, During the Industrial Age, they absorbed approximately one-third of the cumulative anthropogenic CO_2_ emissions^57^. The mathematical models show that CO_2_ injected into the ocean could last for centuries^56^. These cold (approximately 1°C) and deep (around 4 to 5 km) waters move slowly and can remain separate from the atmosphere for millennial time scales. Direct dissolution of CO_2_ in seawater is the primary proposed approach to ocean storage. Liquid CO_2_ shall be discharged directly to the bottom of the sea in the first approach, forming a series of rising droplet columns. On the other hand, liquid CO_2_ is injected into a column in which it reacts with seawater to form hydrates under controlled conditions^58^.

#### Mineral carbonation

The concept of mineral carbonation of CO_2_ (mineralization) as an alternative strategy for CO_2_ sequestration was first introduced by Sifrits^59^. This approach segregates the sequestered CO_2_ through a mineralization procedure, wherein CO_2_ engages in chemical reactions with oxides or hydroxides of alkaline earth metals, such as those found in calcium and magnesium-rich minerals, to form stable carbonate compounds, as illustrated in Reactions 1 and 2. There are two principal strategies for mineral carbonation: in situ and ex-situ. The in situ strategy involves the creation of carbonates by pumping CO_2_ into subsurface rock formations, while the ex-situ strategy refers to the generation of carbonates using pre-mined or locally sourced minerals in a controlled, above-ground industrial set-up. The in situ process pumps CO_2_ directly into underground formations prompting the natural formation of carbonates. On the other hand, the ex-situ process takes place within industrial facilities at the surface, leveraging either mined or accessible minerals to synthetically create carbonates^60,61^.

1 $$CaO_{\left( s \right)} + CO_{{2_{(g)} }} \to CaCO_{{3_{(s)} }} , \quad \Delta H = - 179\,{\text{KJ}}\,{\text{mol}}^{ - 1}$$

2 $$MgO_{\left( s \right)} + CO_{{2_{\left( g \right)} }} \to MgCO_{{3_{\left( s \right)} }} ,\quad \Delta H = - 118\,{\text{KJ}}\,{\text{mol}}^{ - 1}$$

According to the report of Vatalis et al., cheap and efficient CCS methods are obtained through new physicochemical methods in which CO2 adsorption is enhanced based on adsorption in zeolite pores or depleted lignite matrices^62^.

Field examples of carbon dioxide storage by storage type

- EOR
  - Petra Nova Carbon Capture (TX, USA)
  - Abu Dhabi CCS Project (Abu Dhabi, UAE)
  - Uthmaniyah CO2EOR Demonstration (Eastern Province, SAU)
  - Lula (BR)
- Saline aquifers
  - Illinois Industrial Carbon Capture and Storage (IL, USA)
  - Quest (AB, CAN)
  - Tomakomai (JP)

### Hydrogen storage

**Figure 1:**
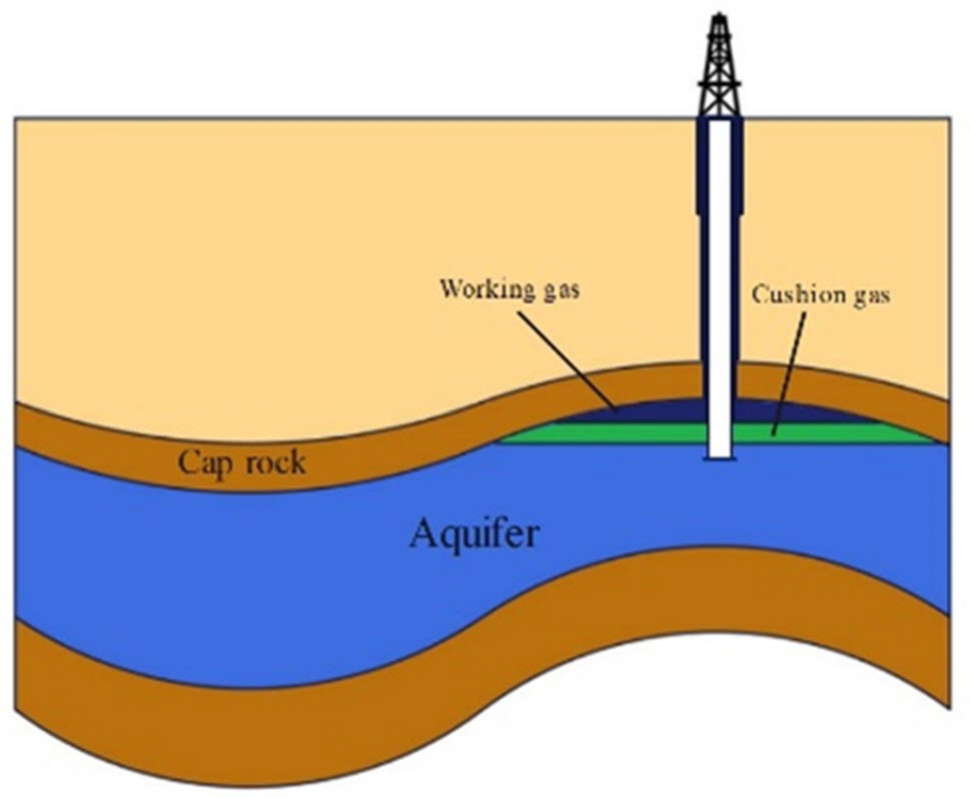
Hydrogen storage in depleted reservoirs.

Hydrogen must be packed, moved, stored, and delivered from production to end use as any other form of product. Investigations are underway to perfect materials for hydrogen storage that are not only secure and dependable but also space-efficient and economically viable for use in fuel cell systems. Similar to other commodities, hydrogen requires encapsulation, movement, containment, and conveyance from the point of manufacture to the end-user. If specific materials are available, Hydrogen can be stored using chemical storage processes or physical absorption. The schematic representation of hydrogen storage is shown in Fig. 1^63^.

#### Chemical storage

Hydrogen storage using chemical methods is a method that draws on techniques where hydrogen production takes place through the process of synthesis. Some substances such as ammonia and LOHCs are used in this method.

Ammonia can be modified for hydrogen production without harmful waste or it can be effectively burned by blending it with existing fuels^64^. The advantage of this material is that not releasing CO_2_ emissions. Advancement in the secure containment of ammonia is progressing through the creation of metal-amine compounds^65^.

##### Metal hydrides

**Figure 2:**
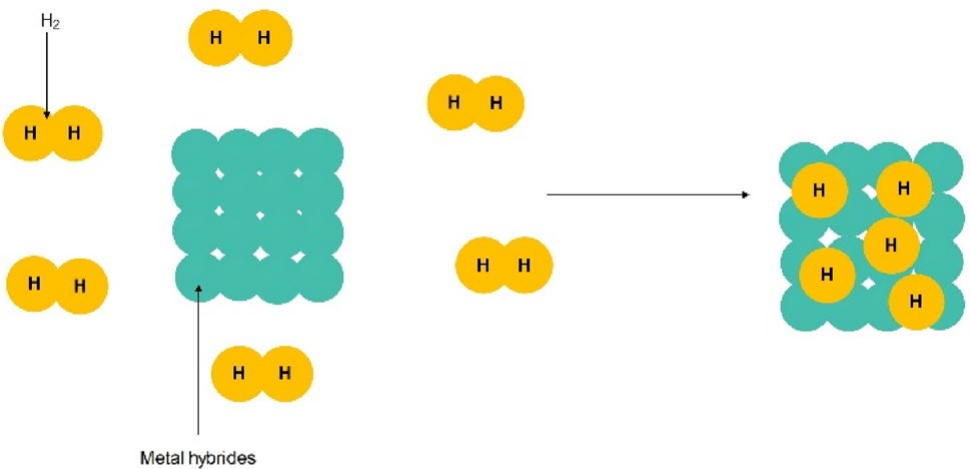
Schematic of hydrogen absorption by metal hydrides.

Metal hydrides possess a distinct capability to store hydrogen and can discharge it at ambient temperature or upon heating the storage vessel. They can store hydrogen up to 5–7% of their weight, but only when heated to temperatures of 2500 °C or higher. These hydrides exhibit a high attraction to hydrogen and necessitate temperatures between 120 to 200 °C to release their contained hydrogen. The metal hydrides selected for storage applications have low reactivity (high safety) and high hydrogen storage density. Metal hydrides possess a distinct characteristic whereby they can uptake hydrogen and subsequently discharge it on-demand, achievable at ambient temperatures or upon the application of heat to the storage vessel. These compounds can typically store hydrogen at a concentration of 5–7% by mass, yet this storage potential is accessible predominantly at elevated temperatures, starting from 2500 °C. Moreover, they exhibit a notable propensity to bond with hydrogen and necessitate temperatures ranging from 120 to 200 °C to liberate the stored hydrogen (Fig. 2). The chosen metal hydrides for storage solutions are characterized by their low level of reactivity (ensuring greater safety) and a high density of hydrogen storage The schematic of metal hydrides is shown in Fig. 2^9,66,67^.

##### Formic acid

**Figure 3:**
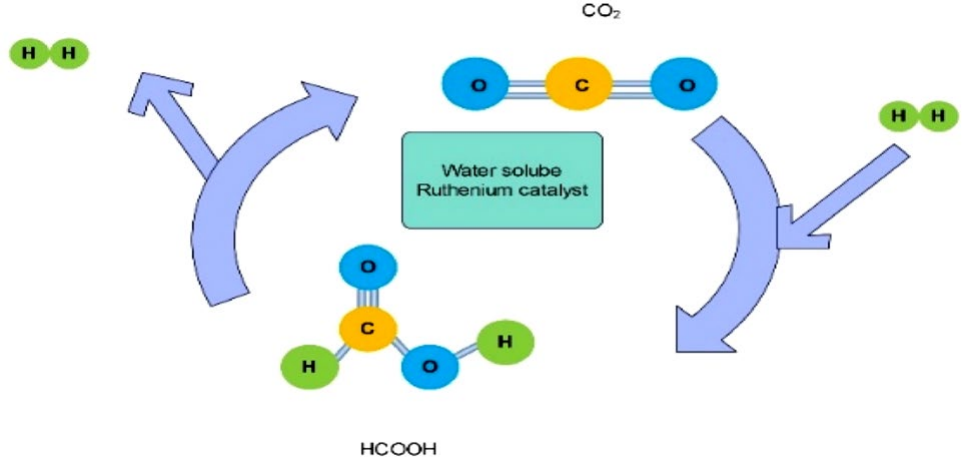
Decomposition of H_2_ and CO_2_ in an aqueous solution through HCOOH.

Given that the hydrogen produced from the reaction does not contain carbon monoxide, there is research interest in utilizing formic acid as a hydrogen storage material. The reaction employs ruthenium catalysts that are dissolved in water, which selectively decompose formic acid into hydrogen and carbon dioxide in an aqueous solution. The stability and lifetime of the catalyst, together with the removal of CO, are improved by applying pressure between 1 and 600 bar, resulting in a durable hydrogen storage material. Carbon dioxide, a typical byproduct of industrial processes, can be repurposed as a hydrogen carrier by hydrogenating it to form formic acid. Scientists are exploring the potential of formic acid for hydrogen storage due to the purity of hydrogen released—devoid of carbon monoxide contamination—during decomposition. This process utilizes ruthenium catalysts that are water-soluble, which selectively break down HCOOH into H_2_ and CO_2_ within an aqueous medium. By exerting pressure within the range of 1 to 600 bar, enhancements in catalyst robustness and operational lifespan are achieved, alongside the elimination of CO, thereby converting it into a more enduring hydrogen storage substance. Additionally, carbon dioxide, a prevalent secondary output in this breakdown procedure, can be repurposed as a hydrogen transporter by its hydrogenation back into formic acid^68–70^. This process is shown in Fig. 3.

##### Carbohydrates

Carbohydrates are the most plentiful renewable biomass resource and offer a high-density storage option for hydrogen in liquid form, achievable under reduced pressure and temperature conditions. Additionally, they can be preserved in solid form. As a result of its complete conversion and moderate reaction conditions, carbohydrates can act as high energy density hydrogen carriers with their polymeric structure evidenced by the formula C6H10O5, making them one of the most abundant sources of renewable biomass. They possess a notable hydrogen storage density when kept in a liquid form, which notably does not necessitate high pressure or temperature conditions. Alternatively, carbohydrates can also be maintained in a solid, powdery state. Owing to their capacity for full conversion under moderate reaction circumstances, carbohydrates serve as an efficient, high-energy density medium for H_2_ transport. (14.8% by weight)^63,71,72^.

3 $$C_{6} H_{10} O_{{5_{\left( l \right)} }} + 7H_{2} O_{\left( l \right)} \to 12H_{{2_{\left( g \right)} }} + 6CO_{{2_{\left( g \right)} }}$$

##### Liquid Organic Hydrogen Carriers (LOHCs)

LOHCs, which have a gravimetric storage density of approximately 6% by weight, are unsaturated organic compounds able to store large amounts of hydrogen. Hydrogen energy may be released or absorbed by LOHs, e.g. Nethyl carbazol. LOHCs, which are unsaturated organic compounds, have the remarkable capability to store considerable quantities of hydrogen. They exhibit a gravimetric storage density that is about 6% by weight. Specific LOHCs, such as N-ethylcarbazole, have the dynamic ability to either emit or absorb hydrogen energy in response to demand. This makes them quite appealing for various applications where hydrogen needs to be stored and transported efficiently^73^.

#### Physical adsorption

This is a process in which H_2_ molecules are poorly absorbed by the material's surface. Physical adsorption is one way of improving kinetic storage and maintaining the molecular identity of H_2_ during this process. Physical adsorption occurs when H_2_ molecules are weakly bound to the surface of the substrate material. This method retains the molecular integrity of H_2_ and can enhance the kinetics of hydrogen storage. Porous materials are particularly effective for this purpose and have been the subject of extensive research. The large surface areas of materials used for this method provide ample space for hydrogen adsorption, making them good candidates for efficient hydrogen storage technologies.

##### Advantages of physical adsorption

The method of compressing gas necessitates high starting pressures that can pose safety risks. On the other hand, cryogenic storage for hydrogen compression requires a significant amount of input energy for initial compression. Complex hydrides (such as Mg_2_NiH_4_) are more expensive, sensitive to impurities, have a lower reversible weight capacity, and can be decomposed at higher temperatures.

##### Carbon-based materials

Hydrogen is adsorbed via van der Waals interaction on the surface of carbon, 6 Kjmol. There is a very low volume density of many special carbon materials with large surface areas, e.g. carbon foam, carbon nanotube, carbon aerogel, and Activated Carbons. Fullerenes, on the other hand, need a very high surface area to be able to achieve an adequate density of packaging. Hydrogen adsorption onto carbon structures occurs through van der Waals interactions, typically around 6 kJ/mol. This relatively weak force is sufficient to bind hydrogen to the extensive surface areas presented by various forms of carbon. Numerous unique carbon configurations, such as carbon foam, carbon nanotubes, carbon aerogels, and activated carbon, exhibit these vast surfaces. Despite their impressive surface area, these materials often have very low volumetric densities. Contrastingly, fullerenes, which are spherical, closed-cage carbon molecules, necessitate a significantly high surface area to attain an adequately dense packing of hydrogen molecules. The high surface-to-volume ratio of fullerenes and other carbon allotropes is beneficial for adsorbing hydrogen, but achieving sufficient volumetric storage densities for practical applications is a challenge, often requiring operation under high pressures or at low temperatures to increase hydrogen uptake.

##### Fullerenes

Fullerenes are made up of five or six interconnected rings, and they each play a role in the formation of C60 molecules by means of their twisted structure. Fullerenes, characterized by their pentagonal or hexagonal rings, form a spheroidal geometry that culminates in the C60 molecule. For a metal atom secured to a carbon-based fullerene, the significant difference in electronegativity with C60 propels electron migration from the metal to the C60, rendering the metal atom positively charged. Consequently, these cationic metal ions capture molecular hydrogen via polarization-driven interaction. Nonetheless, theoretical models suggest the metal atom retains significant isolation when positioned on C60. Titanium, in particular, tends to aggregate on C60, and this clustering phenomenon is unrelated to the type of hydrogen bonds present. Such aggregation of titanium, however, detracts from the weight efficiency of hydrogen storage.^74,75^.

##### Carbon nanotubes

**Figure 4:**
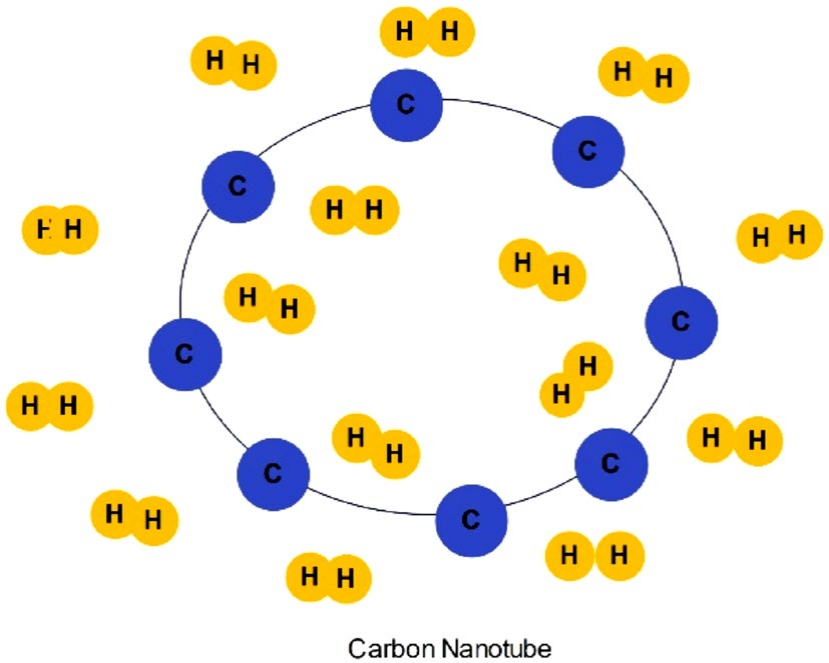
Structure of carbon nanotubes.

Carbon nanotubes (CNTs) are microscopic carbon tubes^76^ with a thickness of about two nanometers. They are capable of containing hydrogen within their minuscule pores or inside the actual structure of the tubes. The structure of carbon nanotubes is depicted in Fig. 4. Nanotubes may exhibit a structure of either a single layer or multiple layers, possess numerous sites for adsorption, exhibit a substantial density of packing, and hold a theoretical weight capability of 6%^74^. Both carbon nanotubes and fullerene-like structures have undergone chemical alterations with transitional or alkaline metals to augment the adherence of H_2_ molecules upon these metal-enhanced carbon nanotube composites^77^.

##### Graphene

**Figure 5:**
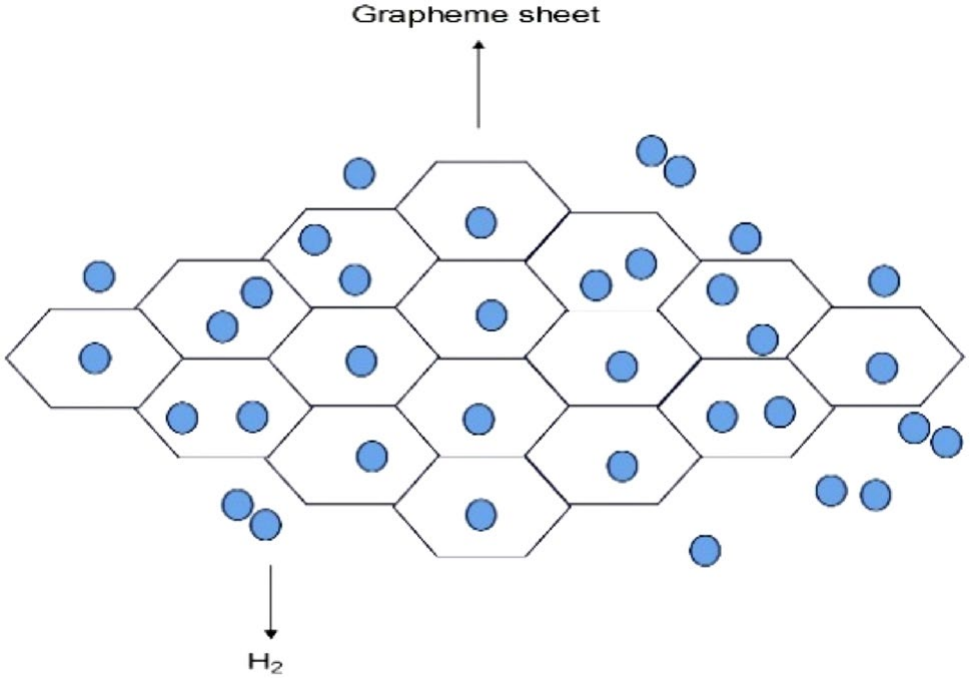
Hydrogen storage in grapheme.

To control the interaction between hydrogen and graphene, it is possible to adjust the distance between adjacent layers, adjust the curvature of the sheet, or use chemical functionalization to control the adsorption and desorption of hydrogen (Fig. 5)^78^. This approach entails trapping hydrogen between graphite layers, which is typically only releasable when heated to approximately 450 °C. It is more efficient compared to carbon nanotubes as it is not only cost-effective but also safe and easy to prepare. The engagement of hydrogen with graphene can be modulated by fine-tuning the spacing amidst neighboring layers, altering the sheet’s curvature, or via chemical modification, which facilitates regulated hydrogen adsorption and release^76^. Employing this technique, hydrogen is retained between graphite layers and is designed to discharge only upon being heated to roughly 450 °C. This approach is recognized as more efficient than the use of carbon nanotubes, given its cost efficiency as well as its safety and simplicity in preparation^79^. The schematic of graphene is shown in Fig. 5.

##### Zeolites

Under high temperatures and pressure, H_2_ is forced to move into molecular sieves with different structures and compositions of the pores in zeolites^80^. When the zeolites cool to room temperature, H_2_ is trapped inside their pores and can be released with an increase in system temperature. Within zeolites, H_2_ is compelled to navigate through the pores of the molecular sieve, which is subjected to elevated temperatures and pressures and possesses a variety of pore structures and compositions^78^. On cooling the zeolites to ambient temperatures, H_2_ is ensnared within these pores and can subsequently be liberated by heating the system. Studies indicate that zeolites featuring sodalite cages have demonstrated an ability to store hydrogen at a capacity of 9.2 cm^3^/g at a temperature of 573 K and a pressure of 10.0 MPa^79^. The high thermal resistance, affordability, and tunable composition of zeolites have led to their acknowledgment in various domains^81^.

##### Metal–organic frameworks

Metalorganic frameworks: MOFs are a family of nanoporous materials consisting of well-defined building blocks, polarized metal oxide centers, and nopolar organic connections. MOFs with oxide components maintain stability even when their pores are vacant or subjected to heating. MOFs exhibit desirable characteristics such as robust stability, high void volumes, well-defined and homogeneously sized cavities, extensive surface areas, adjustable pore sizes, and controllable thermal properties which allow them to stay at an acceptable temperature. MOFs stand as a distinct group of nanoscopic structures characterized by meticulously arranged foundational units, comprising polar metallic oxide junctures (binding points) and apolar organic spans. Even in scenarios where their cavities are vacant or subjected to heat, MOFs with oxide constituents maintain their integrity. These frameworks boast robust constancy, substantial empty spaces, cavities that are consistent in shape and size, extensive surfaces, pores whose dimensions are adjustable, properties that can be modified as needed, and a reliable level of heat resistance^82,83^.

##### Covalent organic frameworks (COFs)

The premier benefit of 3D-COFs, setting them apart from other porous organic materials, stems from their crystalline architecture, facilitating a high surface area. COF-1 and COF-5 epitomize 2D structures, in contrast to COF-102, COF-105, and COF-108, which exemplify 3D configurations—these offer thrice the storage capacity compared to their 2D siblings. Of noteworthy mention is COF-102–3, the only one to present a weight uptake of 26.7% at 77 K and 6.5% at 300 K under a pressure of 100 bar. The primary benefit of 3D-COFs over other porous organic materials of light weight is their crystalline composition, which results in a considerably extensive surface area. COF-1 and COF-5 possess bidimensional formations, in contrast to COF-102, COF-105, and COF-108, all of which feature a tridimensional framework that enables a tripling in storage capabilities when compared to their 2D counterparts. Specifically, COF-102–3 stands out as the singular structure exhibiting significant mass absorption, with 26.7% at the cryogenic temperature of 77 K and 6.5% at the higher temperature of 300 K under the pressure of 100 bar^84^.

##### Micro porous metal coordination materials (MMOMS)

These materials have pore dimensions equivalent to the molecular size of H_2_, making them an efficient storage material for H_2_. Based on aromatic carbon rings, they're composed of open channels. In order to change the curvature of channels, their internal surfaces can be easily changed so as to increase interaction with H_2_ adsorbents. These materials excel at storing H_2_ due to their pore sizes being closely matched to the molecular size of H_2_. Constructed with open pathways formed by aromatic carbon loops, the internal surfaces of these materials are readily adjustable, allowing for alterations in the pathways’ curvature, which enhances the interactions critical for H_2_ adherence^85^.

##### Clathrate

Clathrate hydrates are compounds that can trap molecules within their polyhedral cages; these cages are constructed by water molecules linked through hydrogen bonding. Type I, type II, and H16 are commonly formed into two cubic forms. The crystallographic properties of each structure are different, and the shapes and sizes of the holes are different. Clathrate hydrates consist of compounds that trap guest molecules within their polyhedral enclosures, created by a network of water molecules connected through hydrogen bonds. Typically, they crystallize into two cubic structures, known as type I and type II, as well as a hexagonal variety, type H16. Each of these structural types showcases unique crystallographic characteristics, along with cavities varying in both shape and dimension^86–88^.

##### Glass capillary arrays

The glass capillary arrays are located in the steel tanks which have been designed to withstand pressure. The membranes are secured by melting one end and sealing the other end with an alloy composed of sealing materials. The process of hydrogen addition persists until the desired storage pressure is achieved within the steel container. Currently, glass capillary arrays are employed in mobile applications to ensure the safe injection, storage, and regulated release of hydrogen. Arrays of glass capillaries are fitted within tanks crafted from pressure-tolerant steel. These membrane arrays are subsequently sealed—one end is melted shut, while the opposite end is capped using a sealing metal alloy. Hydrogen is introduced into this assembly until it attains the target storage pressure within the steel vessel. Currently, these glass capillary systems are employed for the secure injection, containment, and regulated discharge of hydrogen, specifically in mobile contexts^89,90^.

##### Glass microspheres

At a temperature of 1 °C, the glass microspheres are initially filled with hydrogen at about 350 to 700 bar pressure. They'll be rapidly cooled to room temperature afterward. For controlled hydrogen release, the spheres shall be transferred to a low-pressure storage vessel and heated again at temperatures of 200–300 °C. These materials not only have low volumetric capacity but also require high-pressure filling. Glass microspheres are subjected to an initial fill of hydrogen at heightened pressures ranging from 350 to 700 bar, and this is conducted at a chilled temperature of about 1 °C. Following the pressurization, they are briskly brought back to ambient room temperature. These spheres are then relocated to a storage vessel with diminished pressure. For the controlled dissemination of the hydrogen, they are reheated to temperatures within the scope of 200–300 °C. While employing these materials, it is worth noting that they are characterized not only by a limited volumetric capacity but also necessitate the application of high pressure during the filling process^91^.

##### Organotransition metal complexes

**Figure 6:**
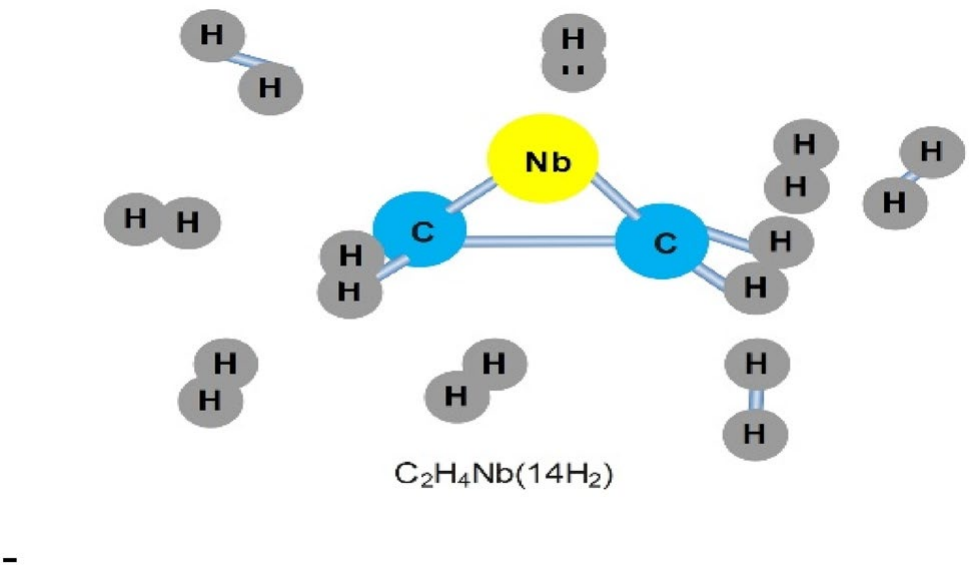
The optimized structure of C2H4Nb(14H2) complex.

Transition metal complexes are compounds with a carbon foundation that encompass intermediate metals in their design, serving to amplify the hydrogen storage capability of the composite structure. Transition metal complexes are composed of carbon frameworks that integrate transition metals, thereby elevating the composite’s capacity to store hydrogen hydrogen. The schematic of the optimized structure of the C_2_H_4_Nb(14H_2_) complex is shown in Fig. 6^92–94^.

##### Theoretical aspects of hydrogen storage

To determine the position at which hydrogen molecules are absorbed into the chemical adsorption system, energy scans have been performed using single-point energy changes as a function of the radial distance of the H_2_ molecule from the center of the H_2_ adsorbent system to the outer surface^95,96^. The dynamic stability significantly influences the efficiency of hydrogen storage, and this theoretically corresponds to the energy difference between the HOMO–LUMO levels of the linked adsorbent group. For optimized H_2_ storage, the dynamic stability of the complex is projected to increase as H_2_ molecules are incrementally added to the metal complex. Variations in single-point energy have been employed to execute energy assessments, reporting them with the gradual outward movement of an H_2_ molecule from the heart of the H2 containment structure to its external limit. This procedure determines the exact point at which hydrogen molecules are genuinely integrated into the chemical containment apparatus^94,95^. Dynamic robustness, which is theoretically related to the energy differential between HOMO–LUMO levels in a Absorbent Group, has an important impact on efficient hydrogen storage. It is expected that the addition of H_2_ molecules in sequence to a metal complex increases the system's dynamic stability and thereby promotes efficient retention of H_2_^95,97^.

By moving towards the use of renewable energies in order to decarbonize and reduce fossil fuels, the use of hydrogen as a source of clean energy becomes important. The properties of hydrogen, including the potential to improve national energy security and fuel economy, boost a country's economy. It also diversifies transportation alternatives for a more flexible system when used to power electric vehicles with highly efficient fuel cells^98,99^.

##### Field examples of hydrogen storage by storage location

- Depleted oil and gas
  o Dladema project (Argentina)
  o Underground Sun (Austria)
- Saline aquifers
  o Ketzin project (Germany)
  o Beynes project (France)
  o Lobodice project (Czech Republic)
- Salt caverns
  o Teesside project (UK)
  o Clemens dome project (USA)
  o Moss bluff project (USA)
  o Spindletop project (USA)
  o Kiel project (Germany)

## Wettability alteration during gas storage

Thermophysical and petrophysical factors including wettability of rock-brine-gas systems and interfacial tension (IFT) between rock and liquids are effective parameters to evaluate the ability to store CO2 and H2 in the reservoir.

One of the influencing parameters on gas storage is rock wettability, which determines fluid mechanics, storage capacity, and rock containment security.

### Wettability measurement

**Figure 7:**
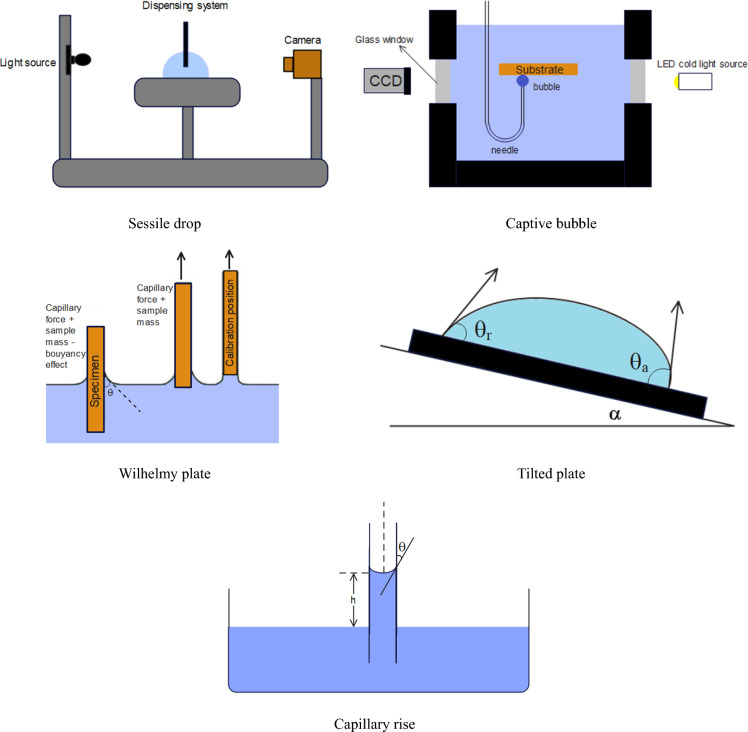
Schematic of contact angle measurement methods^100^.

Usually, measuring wettability is done by measuring the contact angle between solids and fluids. There are various methods for determining the contact angle, including sessile drop, captive bubble, Wilhelmy plate, tilted plate, and capillary rise. The schematic of each of these methods is shown in Fig. 7. The sessile drop method is most commonly used in measuring contact angle values. This method involves placing a liquid droplet on a solid surface and measuring the angle formed between the tangent to the droplet at the common surface with the solid surface. The contact angle provides information about surface wettability^100^.

There are challenges while measuring the contact angle parameter, some of which are:

- Surface contamination: Contaminants on the surface can alter contact angle measurements, leading to inaccuracies.
  Resolution: Implement thorough surface cleaning protocols using appropriate solvents and techniques. Utilize clean substrates and ensure proper handling to minimize contamination^101,102^.
- Substrate heterogeneity: Variations in substrate properties can introduce inconsistencies in contact angle measurements.
  Resolution: Select substrates with uniform surface properties and minimal heterogeneity. Conduct surface characterization to identify and mitigate any inherent substrate variations^103,104^.
- Dynamic nature of gas dissolution: Gas dissolution can lead to time-dependent changes in surface properties, affecting contact angle measurements.
  Resolution: Employ dynamic contact angle analysis techniques to capture changes over time. Conduct experiments under controlled environmental conditions to minimize variability^105^.
- Instrumental limitations: Inaccuracies in measurement instruments or techniques can impact the reliability of contact angle data.
  Resolution: Use high-precision instruments with appropriate calibration and validation procedures. Follow standardized measurement protocols to ensure consistency and reliability^106^.
- Gas adsorption: Gas molecules can adsorb onto the substrate surface, altering its properties and influencing contact angle measurements.
  Resolution: Minimize gas adsorption effects by conducting experiments in controlled atmospheres or vacuum conditions. Utilize appropriate surface treatments or coatings to mitigate gas adsorption^107^.
- Interfacial instabilities: Rapid changes in gas composition or pressure can induce interfacial instabilities, affecting contact angle measurements.
  Resolution: Control gas composition and pressure gradients during experiments to minimize interfacial instabilities. Employ stabilization techniques or additives to maintain interface integrity^108^.

### Wettability alteration during carbon dioxide storage

In 2007, Chiquet et al. investigated the contact angle of brine and CO_2_ as fluid on mica and quartz as solid surfaces at a pressure range of 1 to 11Mpa and salinities of 0.01, 0.1, 0.2, 0.5, and 1 M of NaCl and a temperature of 35°C. The results demonstrated a transition from a water-wet state at low pressures to an intermediate water-wet state at pressures higher than 10 MPa. This change was more pronounced for mica compared to quartz^109^.

**Figure 8:**
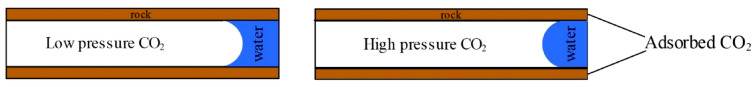
Wettability alteration caused by absorption of CO2 on rock surface.

At high pressures, the contact angle of water increases with the absorption of CO_2_ onto the rock surface. This process is illustrated in Fig. 8.

In 2017, a study conducted by Al-Anssari et al. investigated the impact of pressure (0.1 to 20 Mpa), temperature (23, 50, and 70 °C), silica concentration(0.01, 0.05, and 1%wt,) and treatment duration (0.5 to 4 h) and salinity (0 to 20%wt of NaCl and CaCl2) on wettability. Through nanoparticle treatment, they were able to transform the wettability of calcite from an intermediate wet state to strongly water-wet. Furthermore, they noted that the reduction in water contact angle due to nanoparticle treatment became less pronounced at higher treatment temperatures. Conversely, the decrease in water contact angle caused by CO_2_ pressure showed an upward trend across all temperature levels. The contact angle experienced a decline with both time and nanoparticle concentration. The study also established that under normal pressure conditions, the optimal pH range for desirable wettability was between 4 and 6. However, when subjected to elevated CO_2_ pressure, a pH range of 6 to 8 exhibited the most significant effect on surface wettability. Generally, the presence of NaCl did not substantially alter the contact angle of natural calcite. On the contrary, the introduction of CaCl_2_ led to a noticeable reduction in water contact angle. On the contrary, an increase in pH corresponds to a decrease in the contact angle of calcite treated with nanospheres. In particular, the effects of divalent ions were more pronounced than those of NaCl^110^.

In 2021, the study by Fatah et al. delved into the influences of time (3, 5, 7, 9, 11, 13, 15 at 18Mpa and 70°C), temperature (40, 50, 60, 70, 80, and 90°C at 10Mpa for 48 h), and pressure (9, 12, 15, 18, 24Mpa at 10°C for 48 h) on wettability. The findings revealed that shale formations rich in clay could undergo alterations in their CO_2_-wet characteristics as treatment time and pressure increased. Conversely, shales with significant quartz content retained their robust hydrophilicity. Raising the temperature heightened the interaction between shale and CO_2_, with a minor effect on shale wettability. Moreover, an augmentation in cohesive energy density led to a decrease in surface hydrophilicity^111^.

In 2023, Lu et al. conducted a study focused on modifying wettability within water/shale/CO_2_ systems through the application of nanoparticles with dosages of 0.025, 0.05, 0.1, 0.2, and 0.4%wt. In the study, the substrates were divided into nanofluid-treated and nanofluid-untreated groups. The treated group underwent ScCO_2_ treatment (4 days/16 MPa/40°C) to reduce hydrophilicity, followed by immersion in nanofluids at different times and concentrations. They were then dried at 60°C for 24 h and exposed to ScCO_2_ again. The untreated group was continuously exposed to ScCO_2_ (8 days/16 MPa/40°C). The introduction of supercritical carbon dioxide led to an increase in the water contact angle within treated samples. Nevertheless, the utilization of nanofluids had the potential to reverse this effect. The research indicated that in this reversion process, silica nanofluids displayed more effective outcomes compared to alumina nanofluids^112^.

In 2021, Iglauer and Al-Yaseri presented a solution to address challenges related to CO_2_ storage by suggesting the application of anionic surfactants on basalt surfaces at pressures of 5 to 15Mpa at 308.15, 323, and 333.15K and a NaCl concentration of 0.3M. Their approach involved the use of sodium dodecylbenzene sulfonate. The experimental findings strongly underscored the success of this technique. Notably, even when subjected to elevated pressures and low concentrations of sodium dodecylbenzene sulfonate, basalt exhibited completely water-wet behavior. This outcome highlighted the efficacy of their proposed strategy^113^.

In 2022, Al-Yaseri et al. explored the influence of organic acids on the wettability of calcite, mica, and quartz minerals. The findings unveiled a consistent pattern wherein the ascending order of mineral hydrophobicity was calcite > mica > quartz across all conditions. At a temperature of 323K and a pressure of 25 MPa, when stearic acid was present at a concentration of 10^–2^ mol/l, quartz underwent a transition to an intermediate wet state. In contrast, mica and calcite demonstrated CO_2_-wet behavior under the same conditions^114^.

In 2024, Sakthivel et al. conducted an exploration into the impact of carbon nanodots on the wettability of carbonate rock/brine/CO_2_ systems. This investigation encompassed diverse concentrations of carbon nanodots (0–1000 ppm), varied temperatures (20–80°C), and pressures (14.7–3000 psi). The study unveiled that even following treatment with carbon nanodots, the carbonate rock maintained a robust water-wet nature. Upon elevating the pressure to 1000 psi, across all temperatures, the initially oil-wet carbonate samples underwent a shift toward CO_2_-wet behavior. Interestingly, when subjected to 3000 psi pressure and treated with carbon nanodots, the carbonate samples transformed into a mildly water-wet state. Generally, the trend observed indicated that an increased concentration of carbon nanodots correlated with a decline in the contact angle. However, with the augmentation of both temperature and pressure, the contact angle exhibited an upward trend^115^.

Due to the dissolution of carbon dioxide in the formation water, carbonic acid is generated, impacting the wettability values. This phenomenon was explored by Drexler et al. in 2020 at conditions of 60°C and 6.895 MPa. Their findings revealed that carbonated brine lowers the pH of the aqueous phase, facilitating the protonation of acidic and basic compounds in the oil. This elevation in positive charge at the oil/brine interface leads to enhanced repulsion with the positively charged carbonate rock surface. Consequently, there is a shift towards water-wet in terms of wettability^116^.

Contact angle values were measured by Al-Yaseri et al. for systems containing nitrogen and CO_2_ and a mixture of these two gases (50% mol). The values of temperature, pressure, and salinity of the system were equal to 333 K, 13 MPa, and 5000 ppm NaCl, respectively. All systems were weakly water-wet and had advancing contact angles equal to 47, 40.6, and 33.9 for pure CO_2_, pure N_2,_ and the combination of these two gases^117^.

The effect of SO_2_ impurity on the wettability of a CO_2_/brine/quartz system in 2014 investigated by Saraji et al. in the temperature range of 50 to 100 °C, pressure between 2000 and 4000 psig and salinity between 0.2 and 5 M, and weight percent of SO_2_ between 0 to 6 M. Based on this, increasing the pressure caused a slight increase in the contact angle of advancing and receding. Both the advancing and receding contact angles of water increased with increasing salinity, but no type of contact angle changed with increasing SO_2_ concentration. All systems were strongly water-wet^118^.

In 2021, Yong et al. investigated the contact angle of water in a CH_4_/CO_2_/graphite system at a temperature of 300 K and a pressure of 5.36 MPa. Accordingly, with the increase in CO_2_ concentration, while all systems had weak gas-wet properties, the water contact angle increased^119^.

The wettability of microcline, quartz, and illite at pressures of 2 to 25 MPa and temperature of 40 °C and salinity of 5.19 M was investigated by Botto et al. in 2017. By increasing the pressure from 2 to 7.38 MPa (transition to supercritical), the contact angle values increased strongly. But after that, according to the error values, it can be said that the contact angle remains constant. All samples had strongly water-wet behavior at all pressures^120^.

The wettability changes of a scCO_2_-silica-brine system at a pressure of 8.5 MPa and a temperature of 45°C were investigated in different salinities from 0.01 to 5M of NaCl by Kim et al. in 2012. The contact angle of brine increased from values close to 0° to 80° with a larger increase in higher ion strength conditions^121^.

In 2014, Shojai Kaveh et al. measured the contact angle of the CO_2_/water/benthemier sandstone system at 45°C and pressures of 0.2 to 15 MPa. Accordingly, by increasing the pressure from 1 to 9.2 MPa, the contact angle reached from 15° to 20.5°. After increasing the pressure to 12.8 MPa, the contact angle decreased. In all pressures, the rock kept its strongly water-wet property^105^.

Table 1 shows the effect of different parameters on the wettability altrations during the injection of CO_2_.

**Table 1:** The effect of different parameters on wettability changes in the presence of CO_2_.

References	Temperature (K)	Pressure (MPa)	Mixture(s)	Findings
Chiquet et al.^109^	308.15	1–11	CO_2_	Decrease in water wettability at higher pressures More change for mica compared to quartz
Kim et al.^121^	318.15	8.5	CO_2_	The contact angle of brine increased from values close to 0° to 80° with a larger increase in higher ion strength conditions
Shojai Kaveh et al.^105^	318.15	0.2–15	CO_2_	Slight increase in contact angle by increasing pressure from 1 to 9.2 MPa The contact angle decreased by increasing pressure to 12.8 MPa
Saraji et al.^118^	323.15–373.15	13.79–27.58	CO_2_ + 0–6 M SO_2_	Slight increase in contact angle with increase pressure and salinity No wettability alteration by using SO_2_
Al-Yaseri et al.^117^	333	13	CO_2_ N_2_ 50 mol% CO_2_ + 50 mol% N_2_	Pure CO_2_ showed the highest contact angle The N_2_-CO_2_ mixture showed the lowest contact angle
Al-Anssari et al.^110^	296.15, 323.15, and 343.15	0.1–20	CO_2_	Increase in water wettability with using nanoparticles Decrease in contact angle with time and NP concentration Optimal pH of 4–6 at ambient condition Optimal pH of 6–8 at high pressure Decrease in contact angle in the presence of NP with salinity
Botto et al.^120^	313.15	2–25	CO_2_	Strong increase in contact angle with pressure to supercritical and no change after that
Drexler et al.^116^	333.15	6.895	CO_2_	Carbonic acid shifts rock wettability to a water-wet state
Fatah et al.^111^	313.15, 323.15, 333.15, 343.15, 353.15, and 363.15	9, 12, 15, 18, and 24	CO_2_	Shift to CO_2_-wet with time and pressure in shales rich in clay Shales with quartz remained strongly water-wet Minor effect of temperature on wettability
Iglauer and Al-Ansari 2021^113^	308.15, 323, and 33.15	5–15	CO_2_	Decrease in contact angle even at low dosage of sodium dodecylbenzene sulfonate
Yong et al.^119^	300	5.36	CO_2_ CH_4_ CO_2_ + 20 to 80% CH_4_	Contact angle decreased by introducing CH_4_
Al-Yaseri et al.^114^	323 K	25	CO_2_	Quartz shifts to intermediate wet in the presence of stearic acid Mica and calcite are CO_2_-wet in the same condition
Lu et al.^112^	313.15	16	CO_2_	Increase in contact angle with ScCO_2_ Using nanofluids decreased the contact angle Silica was more effective than alumina
Sakthivel et al.^115^	293.15–353.15	0.1–20.68	CO_2_	Strongly water-wet state with using nanodots Shift from oil-wet to CO_2_-wet with pressure Decline in contact angle with nanodots concentration

### Wettability alteration during hydrogen storage

Iglauer et al. conducted research in 2021 to explore the influence of temperature (296–343 K), pressure (0.1–25 MPa), and the concentration of organic acid (10^–2^, 10^–3^, 10^–5^, 10^–7^, and 10^–9^ mol/L) on the wettability of quartz rock by hydrogen. The findings indicated that, in an actual storage setting, an escalation in temperature, pressure, and organic acid concentration resulted in an increased hydrogen wettability^122^.

In 2021, Al-Yaseri and Jha conducted a study examining the wettability of brine/gas/basalt systems at 323K and four distinct pressure levels (5, 10, 15, and 20 MPa). The findings indicated that basalt maintains its strong hydrophilic characteristics when exposed to hydrogen under storage conditions^123^.

In 2021, Ali et al. conducted a study examining the influence of organic acids on the wettability of quartz at 323K and under three different pressure levels: 0.1 MPa, 15 MPa, and 25 MPa. Their findings indicated that when the sample was exposed to organic acids with longer chains, the quartz surface exhibited a strongly water-wet behavior. However, in the presence of hydrogen at 25 MPa and 323K, it transitioned to intermediate water wet. In contrast, under the same conditions, other acids had either a moderate or limited impact^124^.

In 2022, Hosseini et al. conducted a study examining the influence of various factors on the contact angle of the water/hydrogen/calcite system. These factors included pressure (0.1-20MPa), temperature (298–353 K), salinity (0–4.95mol/Kg), stearic acid concentration (10^–9^-10^–2^ mol/L), tilting plate angle (0 to 45°), and surface roughness (341 nm, 466 nm, and 588 nm). Their findings revealed that raising the system's pressure shifted it from being strongly water-wet to an intermediate water-wet state. Furthermore, an increase in stearic acid concentration resulted in a notable rise in the water contact angle, causing the rock surface to become H_2_-wet. Conversely, the contact angle decreased as surface roughness increased. Elevating both salinity and the tilting plate angle contributed to an augmentation in the contact angle. Conversely, an increase in temperature led to a decrease in the contact angle. In conclusion, the study identified optimal conditions for hydrogen storage, characterized by high temperature and pressure, low salinity, and low organic surface concentration^125^.

In a 2022 study conducted by Hosseini et al., they examined the contact angles within the basalt/hydrogen/brine system across varying temperature (308–343 K) and pressure (5–20 MPa) conditions, both without and with the inclusion of organic substances (10^–9^ to 10^–2^ mol/L). The study's findings illustrated that this system displayed a high degree of water-wettability under low-pressure conditions but shifted towards a weaker water-wet behavior as pressure levels increased. Furthermore, the system underwent a transition to an intermediate water-wet state with an increase in the concentration of organic acids and temperature^126^.

In the year 2022, Mukainah et al. conducted an investigation into the impact of total organic content and pressure (14.7–1000 psi) at 50°C on the wettability of the brine/hydrogen/shale system. Their findings revealed that, under atmospheric pressure, the Eagle Ford shale, boasting a substantial total organic content of 3.83%, exhibited complete hydrophobic behavior, whereas the Wolfcamp shale, characterized by a low total organic content of 0.3%, achieved a state of weak water-wettability. Furthermore, it was noted that contact angle values experienced a slight reduction with increasing pressure, suggesting that the shale's hydrogen wettability might not necessarily intensify with pressures up to 1000 psi^127^.

**Figure 9:**
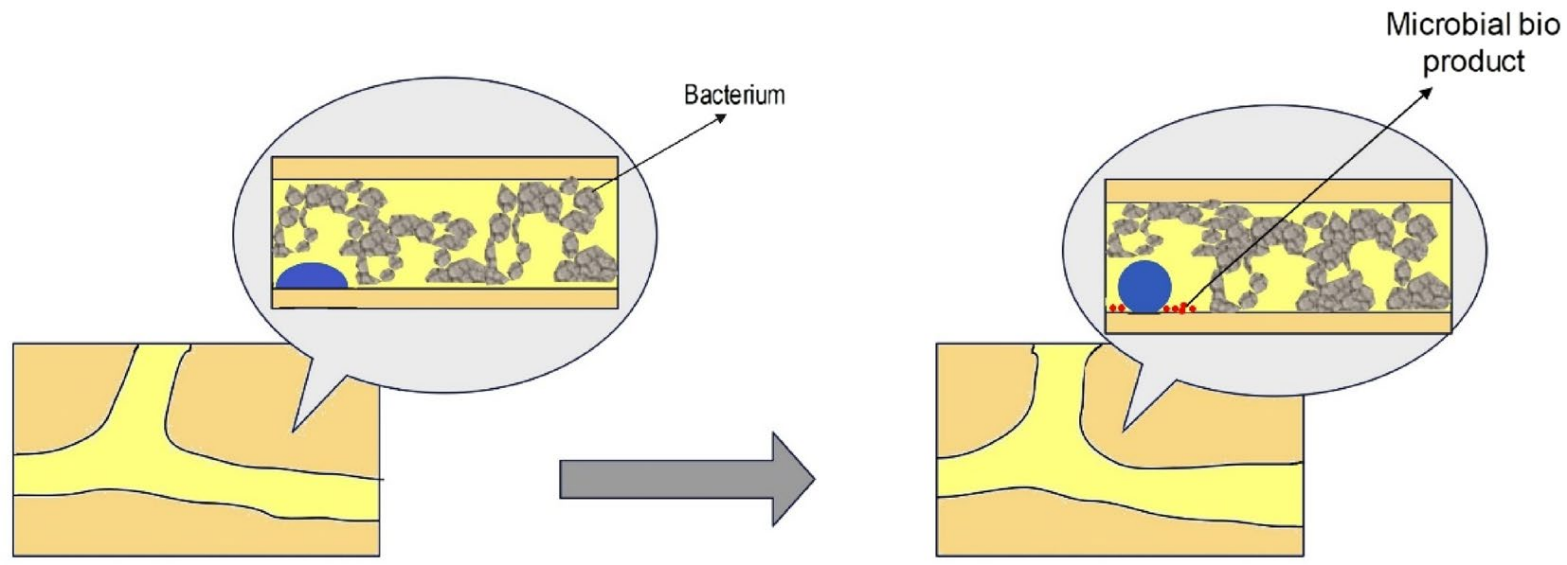
The change in surface wettability in the presence of hydrogen and sulfate-reducing bacteria.

In the year 2023, Liu et al. conducted a study investigating microbial-induced wettability alteration. They focused on a halophilic sulfate-reducing bacterium growing within a microfluidic pore network saturated with hydrogen gas under specific conditions: a pressure of 35 bar and a temperature of 37 °C. The research team assessed changes in wettability by measuring the contact angle within the three-phase system. Their findings revealed that the presence of bacterial cells brought about a shift in wettability, transitioning it from its initial water-wet state to a state characterized as neutral-wet. Conversely, in the sterilized control experiment, no discernible change in the contact angle was observed. This process is shown in Fig. 9^128^.

In 2023, Hosseini et al. conducted a study investigating the influence of silica nanofluids with dosages of 0.1, 0.25, and 0.5 wt% and organic acids on the wettability of Indiana limestone, aiming to optimize it for hydrogen storage. The study explored the effects of various organic acids with differing carbon chain lengths under two different sets of temperature and pressure conditions (298 K and 0.1 MPa, 323 K and 8.27MPa). Additionally, the research examined the impact of silica nanofluids at various concentrations. When the limestone samples were treated with organic acids, a notable trend emerged wherein the contact angle increased as the number of carbon atoms in the acids increased, ultimately transitioning the surface to a hydrophobic state. In contrast, when silica nanofluids were introduced into the experiments, it was observed that the hydrophobicity decreased after the use of these nanoparticles^129^.

In 2023, Zeng et al. carried out a study to investigate how temperature ( 296, 323, and 343 K), pressure (0.1–25 MPa), and varying concentrations of organic acids (10^–9^-10^–2^ mol/L) influenced the wettability of hydrogen on quartz surfaces. Their findings revealed that elevating the temperature and pressure had minimal effects on hydrogen wettability. However, as the concentration of organic acids increased, it correspondingly increased the contact angle of brine, indicating an enhancement in hydrogen wettability^130^.

In 2022, the contact angle of systems containing pure methane, pure hydrogen, and a mixture of these two gases by Hashemi et al. on Bentheimer sandstone at temperatures of 30 and 50°C, pressures of 20, 50, 70, and 100 bar in the presence of distilled water and salinities of 5000 and 50,000 ppm of NaCl was measured. No dependence between salinity, temperature, and pressure with contact angle values was observed in these systems. Also, all gas mixtures had a contact angle between 25 and 45° (strongly water-wet) and it was shown that pure hydrogen, pure methane, and their mixtures have similar wetting properties in real fields^131^.

Table 2 shows the effect of different parameters on the wettability alterations during H_2_ storage.

**Table 2:** The effect of different parameters on wettability changes in the presence of hydrogen.

References	Temperature (K)	Pressure (MPa)	Mixture(s)	Findings
Iglauer et al.^122^	296–343	0.1–25	H_2_	Increase in hydrogen wettability with all three parameters
Al-Yaseri and Jha^123^	323	5, 10, 15, and 20	H_2_	Maintaining the strong water-wet properties of basalt
Ali et al.^124^	323	0.1, 15, and 25	H_2_	Decrease water-wettability in the presence of long-chain organic acids
Hosseini et al.^125^	298–353	0.1–20	H_2_	Decrease in water wettability with pressure increasing the contact angle of water with stearic acid concentration increasing the contact angle with salinity and tilting plate angle decreasing the contact angle with the temperature
Hosseini et al.^126^	308–343	5–20	H_2_	Reduction of water wettability by pressure temperature and organic acids
Al-Mukainah et al.^127^	323.15	0.1–6.89	H_2_	Reduction of contact angle with pressure greater wettability change in shale with higher TOC
Zeng et al.^130^	396, 323, 343	0.1–25	H_2_	Insignificant effect of temperature and pressure on wettability increase in the contact angle of water with an increase in organic acid concentration
Hashemi et al.^131^	303.15 and 323.15	2, 5, 7, 10	CH_4_ + 0 to 80% H_2_	No dependence between salinity, pressure, and temperature with the contact angle Pure H_2_ and pure N_2_ and the mixture had similar wettability states (strongly water-wet)
Liu et al.^128^	310.15	3.5	H_2_	Decreased water wettability in samples containing bacteria
Hosseini et al.^129^	298 and 323	0.1 and 8.27	H_2_	Increasing the contact angle by increasing the carbon number Increase water wettability by using nanofluids

## Interfacial tension alteration during gas storage

Another key parameter when storing gas is the fluid–fluid surface tension, which affects the fluid distribution at the pore scale and the gas storage capacity in the reservoir.

### Interfacial tension measurement

IFT measurement methods can be divided into 5 categories. A schematic of these 5 groups is shown in Table 3. Group IV methods will be explained in the following.

**Table 3:** Schematic of fluid–fluid IFT measurement methods^132^.

Group	Method	Schematic
I: Direct measurement using a microbalance	Wilhelmy plate	
	Du Nouya ring	
II: Measurement of capillary pressure	Maximum bubble pressure	
	Growing drop	
III: Analysis of capillary gravity forces	Capillary rise	
	Drop volume	
IV: Gravity-distorted drops	Sessile drop	
	Pendant drop	
V: Reinforced distortion of drop	Spinning drop	
	Micropipette	

#### Pendant drop

Using a straightforward approach, two parameters essential for experimental determination in the pendant drop method are the equatorial diameter, denoted as D, and the diameter, labeled as d, measured at a distance D from the top of the drop.

To achieve high-quality and consistent results in interfacial tension measurement, the pendant drop technique, like other methods, demands meticulous cleanliness. Ensuring the needle utilized for suspending the drop is thoroughly cleaned is paramount, while preventing any interface climbing along the needle's outer surface. Needles crafted from stainless steel or glass, known for their ease of cleaning with various agents such as acids, bases, and organic solvents, are commonly preferred in surface chemistry laboratories. It's advisable to employ needles with a diameter less than 0.5 times the equatorial diameter (D) of the drop. However, excessively small needle diameters should be avoided as they diminish the value of d and consequently compromise the precision of interfacial tension determination^132^.

#### Sessile drop

The sessile drop technique aligns with the pendant drop method in its approach to analyzing drop shape. However, unlike the pendant drop method where the drop is suspended from a tube, in the sessile drop method, the drop rests on a solid substrate. The wettability of the solid substrate by the fluid significantly affects the outcome in this scenario. By analyzing shape and distance measurements, the interfacial tension can be determined using Eq. (4) as follows:

4 $$\sigma =\frac{\Delta \rho g{z}_{e}^{2}}{2}$$

where z_e_ is the distance from the equator of the drop to its top^133^.

It should be noted that the challenges mentioned in relation to the contact angle for IFT also exist and should be solved appropriately^134–137^.

### Interfacial tension alteration during carbon dioxide storage

Rock/CO_2_ interfacial tension is an essential factor to understand the interaction between CO_2_ and rocks. Low values of rock/CO_2_ interfacial tension suggest stronger CO_2_-rock interaction, thus lower CO_2_ capacity is inferred, and vice versa^138^.

In 2007, Chiquet et al. conducted a study to explore the influence of pressure (5–45 MPa) and temperature (308–383 K) on CO_2_-water IFT. The findings revealed that as the pressure levels increased, there was a significant decrease in the IFT values. Beyond a certain threshold, approximately exceeding 20 MPa, the IFT values reached a pseudo-plateau which slightly increases with temperature. (transitioning from approximately 30 mN/m at 308 K to 23 mN/m at 383 K). Furthermore, the presence of 20 g/L of NaCl had a negligible impact on the IFT values^139^.

In 2021, Abdulelah et al. conducted a study examining alterations in basalt-CO_2_ IFT within the pressure range of 4 MPa to 20 MPa and temperatures spanning from 308 to 333 K. The study revealed that basalt-CO_2_ IFT declined as pressure increased, yet it did not fall below basalt/brine IFT until the contact angle dipped below 90°. Furthermore, the basalt-CO_2_ IFT exhibited an upswing with rising temperatures, and the solid/brine interfacial energy also increased with higher temperatures. As the contact angle neared roughly 80 degrees or when the pressure reached 17 MPa, the sealing capacity of CO_2_ decreased by up to 50%. Moreover, a noteworthy correlation emerged between the basalt-CO_2_ IFT and the density of CO_2_ at temperatures of 308 K and 333 K^138^.

In 2023, Sakthivel et al. conducted a study with a nanodotes concentration range of 0–1000 ppm and pressures of 14.7–3000 psi at temperatures from 20 to 80 °C, and their findings indicated a modest reduction in the seawater-CO_2_ IFT as the concentration of carbon nanodots increased. Nevertheless, on the whole, it can be concluded that the utilization of carbon nanodots did not exert a substantial influence on the seawater-CO_2_ IFT^115^.

In 2022, Al-Yaseri et al. explored the influence of organic acids on the rock-CO_2_ and rock/brine IFT At a temperature of 323K and a pressure of 25 MPa. Their observations revealed that elevating the concentration of stearic acid led to an augmentation in the rock-brine IFT. Interestingly, alterations in pressure did not impact this particular parameter. In contrast, when organic acids were introduced, the rock-CO_2_ IFT exhibited a decrease^114^.

Yang et al. investigated the changes in IFT in different ranges of temperature (300–331 K) and pressure (0–30 MPa) in the year 2005. Based on the findings of this research, CO_2_-brine IFT values have a direct relationship with temperature and an inverse relationship with pressure^140^.

Bachu and Bennion in 2009 measured IFT values between CO_2_ and brine at pressures of 2–27 MPa, temperatures of 36–125°C, and salinities of 0–334,000 mg/L. The results showed that IFT decreases with increasing pressure. Also, this parameter has a direct relationship with salinity. As the temperature increases, the IFT values also increase^141^.

In 2019, Mutailipu et al. discussed the IFT values between brine containing NaCl and KCl and CO_2_ at temperature (298–373 K), pressure (3–15 MPa), and different salinities (1–4.9 mol/kg). Findings of this research indicated that the IFT parameter has a direct relationship with temperature and salinity and an inverse relationship with pressure^142^.

In order to investigate the effect of CH_4_ on IFT, in 2016 Liu et al. measured this parameter for CO_2_/CH_4_ mixtures in the temperature range of 77 to 257 °F and pressure between 15 and 5027 psi and salinity 0 to 200,000 ppm. they paid. The results of this research showed that the presence of methane increases the amount of IFT and the intensity of this decrease is also dependent on the molar fraction of this gas. Similarly, in a CO_2_/CH_4_-brine system, the amount of IFT also increases with increasing salinity^143^.

The effect of SO_2_ impurity on IFT in a CO_2_/brine/quartz system in 2014 investigated by Saraji et al. in the temperature range of 50 to 100 °C, pressure between 2000 and 4000 psig, and salinity between 0.2 and 5 M and weight percent of SO_2_ between 0 to 6 M. Based on this study, pressure did not have a significant effect on the amount of IFT, but the increase in temperature caused a slight decrease in IFT. The increase in salinity has also increased IFT. Also, increasing the amount of SO_2_ has caused a decrease in IFT^118^.

The values of IFT of CO_2_ in the presence of H_2_S in 2008 were investigated by Shah et al. at three temperatures of 40, 70, and 120 °C and pressure between 0 and 15 MPa and 30% molar H_2_S. Based on the findings of this research, the increase in H_2_S reduces IFT values drastically^144^.

The IFT values of CO_2_-brine, N_2_-brine, and CO_2_/N_2_-brine (50 mol% N_2_) systems were investigated by Al-Yaseri et al. in 2015 at a temperature of 333 K and 13 MPa. Accordingly, the N_2_-brine system had higher IFT values than the other two systems, while the CO_2_-brine and CO_2_/N_2_-brine systems had close IFT values^117^.

Table 4 shows the effect of different parameters on the IFT during the storage of CO_2_.

**Table 4:** Effect of different parameters on IFT in the presence of CO_2_.

References	Temperature (K)	Pressure (MPa)	Mixture(s)	Findings
Yang et al.^140^	300–331	0–30	CO_2_	Inverse CO_2_-brine IFT relationship with pressure and direct relationship with temperature
Chiquet et al.^139^	308–383	5–45	CO_2_	Reduction of IFT with increasing pressure and decreasing temperature insignificant effect of 20g/l of NaCl on IFT
Shah et al.^144^	313.15,333.15, and 393.15	0–15	H_2_S CO_2_ 70 mol% CO_2_ + 30 mol% H_2_S	H_2_S reduces IFT values
Bachu and Bennion^141^	309.15–398.15	2–27	CO_2_	CO_2_-brine IFT decreased with increasing pressure CO_2_-brine IFT had a direct relationship with salinity and temperature
Saraji et al.^118^	323.15–373.15	13.79–27.58	CO_2_ + 0 to 6M SO_2_	Pressure had no significant effect on IFT An increase in temperature caused a decrease in IFT Increasing SO_2_ concentration caused a decrease in IFT
Al-Yaseri et al.^117^	333	13	CO_2_ N_2_ 50 mol% CO_2_ + 50mol%N	N_2_-brine had a higher IFT than CO_2_-brine and CO_2_/N_2_-brine
Liu et al.^143^	298.15–398.15	0.1–34.66	CH_4_ CO_2_ CO_2_ + 10.9 to 89% CH_4_	IFT increases in the presence of methane Mixture IFT increases with an increase in salinity
Mutailipu et al.^142^	298–373	3–15	CO_2_	Increase in CO_2_-brine IFT with an increase in salinity and temperature Decrease in IFT with an increase in pressure
Abdulelah et al.^138^	308–333	4–20	CO_2_	IFT decreases with increasing pressure IFT increases with temperature
Al-Yaseri et al.^114^	323	25	CO_2_	Increase of rock-water IFT with increasing stearic acid concentration no effect of pressure on rock-water IFT decrease of rock-CO_2_ IFT in the presence of organic acids
Sakthivel et al.^115^	293.15–353.15	0.1–20.68	CO_2_	Carbon nanodots had no effect on seawater-CO_2_ IFT
Hosseini et al.^145^	298, 323, and 353	5–20	CO_2_	Increase of calcite-gas IFT with temperature, decrease with pressure, salinity, and concentration of organic acids a slight decrease of calcite-water IFT with temperature

### Interfacial tension alteration during hydrogen storage

In 2022, Al-Mukainah et al. investigated the effect of pressure (14.7–1000 psi) at 50°C on the interfacial tension of hydrogen-brine. The results showed that the IFT values decreased with increasing pressure (decreasing from 63.68 mN/m at 14.7 psi to 51.29 mN/m at 1000 psi pressure)^127^.

In 2022, Hosseini et al. conducted measurements of the interfacial tension of hydrogen-brine at different pressures (2.76–34.47 MPa), temperatures (298.15, 323.15, 373.15, and 423.15 K), and salinities (0, 1.05, 3.15, 4.95 mol/kg). When salinity and temperature were kept constant, the IFT decreased linearly with increasing pressure. Additionally, with increasing temperature, the IFT showed a linear decrease. Furthermore, it was observed that with increasing brine molarity, the IFT linearly increased^146^.

In 2018, Florence chow et al. measured the IFT of systems containing pure hydrogen and a mixture of hydrogen and CO_2_ at temperatures between 298.15 and 448.15 K and pressures between 0.5 and 45 MPa. Accordingly, with the increase in temperature and pressure, the IFT values decrease and the presence of CO_2_ will also decrease the values of this parameter^147^.

In 1957, Slowinski et al. investigated H_2_-water IFT values in the pressure range of 0–11 MPa and temperature of 298 K. Based on the findings of this research, increasing the pressure decreases IFT values^148^.

Table 5 shows the effect of different parameters on the IFT during the storage of H_2._

**Table 5:** Effect of different parameters on IFT in the presence of hydrogen.

Reference	Temperature (K)	Pressure (MPa)	Mixture(s)	Findings
Slowwinski et al.^148^	298	0–11	H_2_	IFT had an inverse relationship with pressure
Chow et al.^147^	298.15 and 448.15	0.5–45	H_2_ 30% CO_2_ + 70% H_2_	IFT values decreased with an increase in temperature and pressure Presence of CO_2_ decreases IFT
Al-Mukainah et al.^127^	323.15	0.1–6.89	H_2_	Decreased H_2_-brine IFT with pressure
Hosseini et al.^146^	298.15, 323.15, 373.15, and 423.15	2.76–34.47	H_2_	IFT decreases with temperature and salinity IFT increases with pressure

## Research gaps and future works

Despite the wealth of knowledge garnered from existing research on wettability and IFT alterations during the storage of hydrogen and carbon dioxide, several notable gaps remain, necessitating further exploration for comprehensive understanding and practical applications. While current studies have provided valuable insights into the behavior of these fluids, particularly in relation to changes in wettability and IFT, there is a clear need for more extensive investigations, especially focusing on emerging materials such as specific nanoparticles. Additionally, the literature reveals a conspicuous dearth of research concerning alterations in IFT in the presence of hydrogen, highlighting a crucial avenue for future inquiry.

To fill the current research gaps, the following topics are suggested for future research:

- IFT measurement for hydrogen and H_2_S mixtures
- Examine IFT for combination of hydrogen and SO_2_
- Investigate IFT for hydrogen in the presence of organic acids
- Analyze the effects of nanoparticles such as nanosilica on IFT levels of hydrogen
- Explore the wettability of hydrogen combined with H_2_S
- wettability measurement for hydrogen when mixed with SO_2_
- Study the contact angle of hydrogen in the presence of nanofluids

## Summary and conclusion

Carbon dioxide is recognized as one of the primary factors causing climate change and global warming. Underground storage of carbon dioxide prevents its release helps preserve the environment and mitigates the effects of climate change. Additionally, hydrogen has emerged as a clean and green energy source with the ability to be stored in various conditions. The storage of hydrogen contributes to increased clean energy production and reduced dependency on fossil fuels. Overall, carbon dioxide and hydrogen storage are key solutions for environmental preservation and clean energy production, aiding in the mitigation of negative impacts of climate change and promoting sustainable development. Surface wettability and interfacial tension (IFT) are two crucial parameters in underground storage and extraction of these gases, and they are of special importance.

In this research, the changes in these two parameters during the storage process of hydrogen and carbon dioxide gases were investigated based on previous studies. According to these studies, the use of nanoparticles and anionic surfactants, as well as carbon nanodots, can increase the wettability of the rock during carbon dioxide injection, while the use of organic acids has the opposite effect. Furthermore, an increase in temperature and pressure leads to a reduction in rock surface wettability. An increase in cohesive energy density results in a decrease in surface wettability. Increasing pressure leads to a decrease in the IFT of brine/carbon dioxide. Changes in salinity and the use of carbon nanodots have little effect on this parameter. Increasing pressure and the presence of organic acids can decrease the rock/carbon dioxide IFT, while temperature increases it. The rock/brine IFT increases with salinity and decreases slightly with temperature. During hydrogen gas injection, the wettability decreases with increasing temperature, pressure, salinity, organic acid concentrations, carbon content, and the presence of sulfate-reducing bacteria, but it increases with surface roughness and the use of nanoparticles. The hydrogen/brine IFT decreases with pressure and temperature and increases with salinity. Additionally, the hydrogen/rock IFT decreases with pressure, temperature, and TOC. With an increase in TOC and salinity, the water/rock IFT increases, while it decreases with temperature. The results of this research can be used in the development of storage and utilization of clean energy as well as in controlling the emission of greenhouse gases.

**Figure 10:**
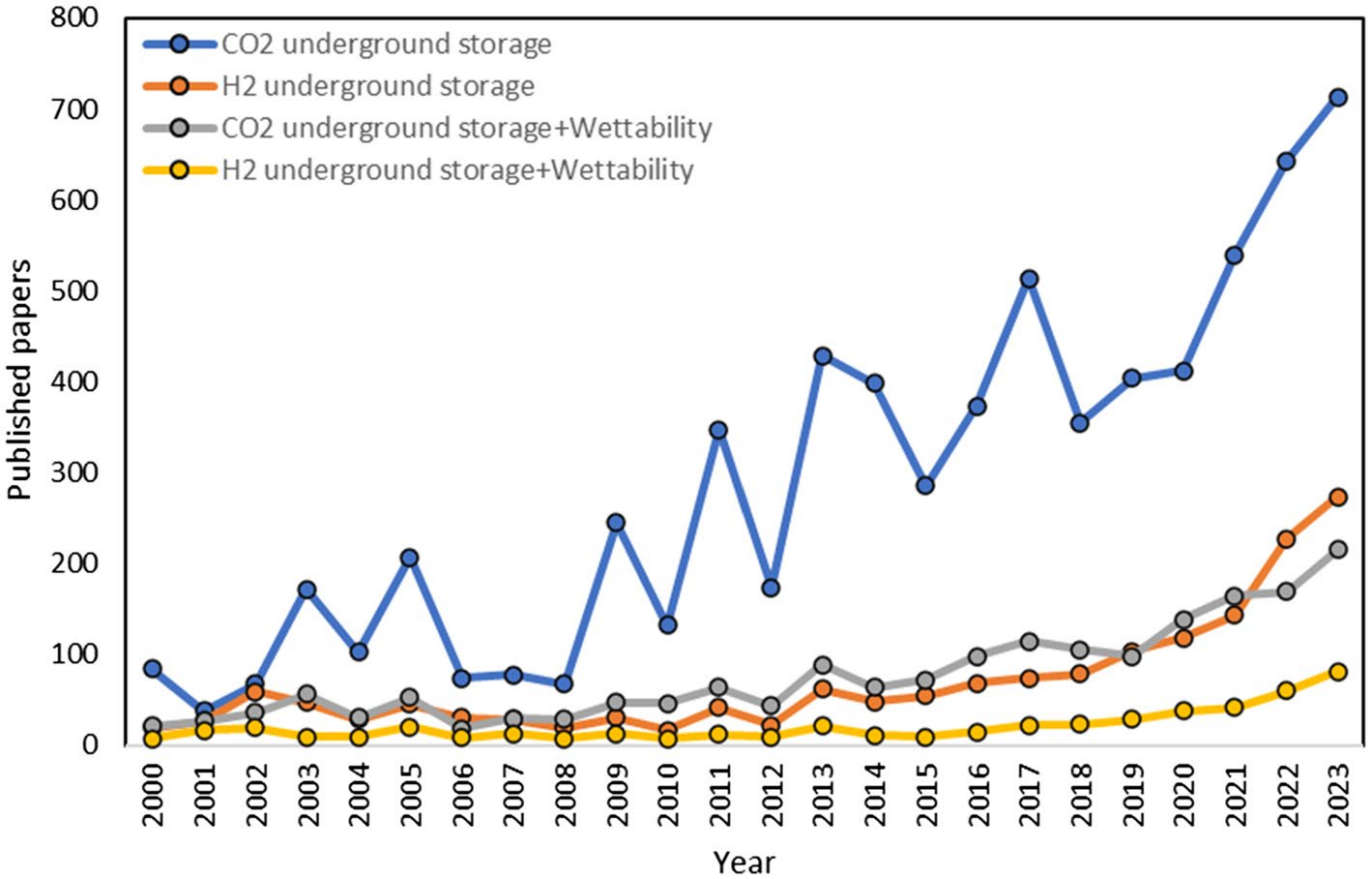
The trend of the number of articles published with different keywords of underground storage of hydrogen and carbon dioxide.

Figure 10 illustrates the trend of published articles in the field of underground storage of hydrogen and carbon dioxide gases and the articles that have focused on wettability during these processes from 2000 to 2023. Based on this figure, studying changes in wettability in the presence of these gases and different influential parameters is of special importance.

## Data Availability

All data generated or analyzed during this study are included in this published article.

## Abbreviations

CBM: Coalbed methane
CCS: Carbon capture and storage
CND: Carbon nanodots
CNT: Carbon nanotubes
COF: Covalent organic frameworks
ECBM: Enhanced coalbed methane
EGS: Enhanced geothermal system
EOR: Enhanced oil recovery
IFT: Interfacial tension
LOHC: Liquid organic hydrogen carriers
MMOM: Microporous metal coordination materials
MOF: Metal organic frameworks
TOC: Total organic carbon
